# Traditional Clinicopathological Biomarkers Still Determine Disease-Free and Overall Survival in Invasive Breast Cancer Patients: A Pilot Study

**DOI:** 10.3390/jcm13072021

**Published:** 2024-03-30

**Authors:** Katarzyna Wrzeszcz, Katarzyna Kwiatkowska, Piotr Rhone, Dorota Formanowicz, Stefan Kruszewski, Barbara Ruszkowska-Ciastek

**Affiliations:** 1 Department of Pathophysiology, Faculty of Pharmacy, Nicolaus Copernicus University, Collegium Medicum, 85-094 Bydgoszcz, Poland; katarzynakwiatkowska@abs.umk.pl; 2 Clinical Ward of Breast Cancer and Reconstructive Surgery, Oncology Centre Prof. F. Łukaszczyk Memorial Hospital, 85-796 Bydgoszcz, Poland; rhonep@co.bydgoszcz.pl; 3 Poznan University of Medical Sciences, Department of Medical Chemistry and Laboratory Medicine, 60-806 Poznan, Poland; doforman@ump.edu.pl; 4 Biophysics Department, Collegium Medicum of Nicolaus Copernicus University, 85-067 Bydgoszcz, Poland; skrusz@cm.umk.pl

**Keywords:** invasive breast cancer, cancer recurrence, prognostic factors, disease-free survival, overall survival

## Abstract

**Background:** Molecular classification, tumor diameter, Ki67 expression, and brachytherapy administration still act as the most potent potential predictors of breast cancer recurrence and overall survival. **Methods:** Over the period of 23 months, we included in the study 92 invasive breast cancer (IBrC) patients initially diagnosed at the Clinical Ward of Breast Cancer and Reconstructive Surgery, Oncology Center in Bydgoszcz, Poland. The probability of disease-free survival (DFS) and overall survival (OS) in relation to potential prognostic factors for the patients were determined using a Kaplan–Meier analysis, and univariate and multivariate Cox regression analyses evaluated the predictive factors of IBrC patients. The investigation of the potential prognostic model’s accuracy was analyzed using the ROC curve. **Results:** Patients with tumor size < 2 cm, Ki67 expression < 20%, luminal-A molecular subtype, and extra-dose brachytherapy boost administration displayed the most favorable prognosis according to breast cancer disease-free survival and overall survival. The estimated 5 year probability of DFS and OS rates in women with tumor diameter < 2 cm were 89% and 90%, respectively. In tumor diameter > 2 cm, the estimated 5 year probability of DFS was 73% and OS was 76%. Interestingly, the tumor diameter of 1.6 cm with a specificity of 60.5% and a sensitivity of 75% occurred as the best threshold point to differentiate patients with cancer recurrence from those without cancer progression. **Conclusions:** Our study provides essential information on the clinicopathological profile and future outcomes of early stage IBrC patients. Furthermore, the tumor diameter cut-off value of 1.6 cm discriminating between disease recurrence and those without disease progression patients represents an innovative direction for further research.

## 1. Introduction

The World Health Organization has identified breast cancer (BrC) as the most prevalent form of cancer worldwide as of 2021. It is estimated to account for approximately 12% of all newly diagnosed cancer cases on an annual basis [1]. The etiology of breast cancer is complex, and its exact pathophysiological pathways are still under elucidation. Generally, breast cancer susceptibility is associated with lifestyle/modifiable (including diet, physical activity, smoking and alcohol status, exogenous hormone exposure) and personal/non-modifiable (including genetic background, age at menarche and menopause, breast density) factors [2,3].

Due to the heterogeneous nature of breast cancer, various treatment strategies and, subsequently, survival rates were established. Based on immunohistochemical evaluation of hormone receptors (including estrogen and progesterone receptors (ER and PR, respectively) and human epidermal growth factor receptor 2 (HER2)) and proliferation marker (Ki67), four molecular (intrinsic) invasive breast cancer subtypes were distinguished, namely, luminal A, luminal B, non-luminal HER2 positive and triple negative. They all require different treatment approaches and vary regarding future outcomes [4]. Of all breast cancer molecular subtypes, luminal A tumors tend to have the best prognosis, whereas luminal B and non-luminal HER2 positive are correlated with poorer clinical outcomes. Triple-negative breast cancer poses a serious challenge for contemporary medicine because of its most aggressive nature and worst survival rates [5]. Indeed, the molecular subtypes of breast cancer play a pivotal role in determining the most appropriate treatment strategy for individual patients. The imperative for molecular classification lies in its ability to stratify patients who stand to benefit from targeted therapies, including hormone therapy and HER2 inhibitors [6]. Patients with luminal A breast cancer often experience favorable outcomes with hormonal therapies such as tamoxifen or aromatase inhibitors. In contrast, luminal B tumors, which may exhibit higher proliferation rates or HER2 overexpression, may benefit from a combination of hormonal therapy and chemotherapy to address their more aggressive nature. HER2-positive breast cancer patients have greatly benefited from targeted therapies such as trastuzumab and pertuzumab [7,8]. Apart from molecular (intrinsic) subtypes, for complete diagnosis, other factors are taken into consideration, including lymph node status, histological type, grade, clinical stage, and menopausal status [9]; these features are also relevant concerning treatment planning. The association between breast cancer treatment and survival rates is a critical aspect of oncology research, as it directly impacts patient outcomes and guides clinical decision-making. Various treatment modalities, including surgery, radiation therapy, chemotherapy, hormonal therapy, targeted therapy, and immunotherapy, are employed either alone or in combination, depending on the characteristics of the tumor and the patient’s overall health status. Understanding how different treatment approaches influence survival rates is essential for optimizing patient care and improving long-term prognosis.

Adjuvant treatment of IBrC consists of breast-conserving therapy (BCT; generally followed by intermediate-dose radiotherapy) and mastectomy, which are both well-known local therapies, or/and systemic therapy, composed of chemotherapy, hormonal therapy, or immunotherapy [10,11]. The primary goal of adjuvant systemic treatment is to reduce the formation of distant metastases. Nevertheless, tumor diameter larger than 2 cm, higher grade, lymph node involvement, and triple-negative subtype are considered as prognostic factors for cancer dissemination, independent of treatment strategy [11,12].

There is a general point of view that early detection of breast cancer is highly curable [4]. Since it is believed that a 5 year survival rate of 99%, 93%, 72%, and 29% for stages I, II, III, and IV, respectively [13], it nevertheless seems that there are additional factors that can influence overall survival at an early stage of tumor development. Thus, looking for an adequate prognostic profile in IBrC is still an unmet oncological need. Despite the advances in medicine and the availability of numerous molecular tests in recent years, the traditional clinicopathological factors still remain essential in determining prognosis and guiding therapeutic decisions for early stage invasive breast cancer patients. This study investigated the association between commonly used biomarkers and disease-free survival and overall survival in the IA-IIB stage cohort of patients with IBrC based on a 6 year follow-up.

## 2. Materials and Methods

### 2.1. Participant Characteristics and Follow-Up

**Figure 1:**
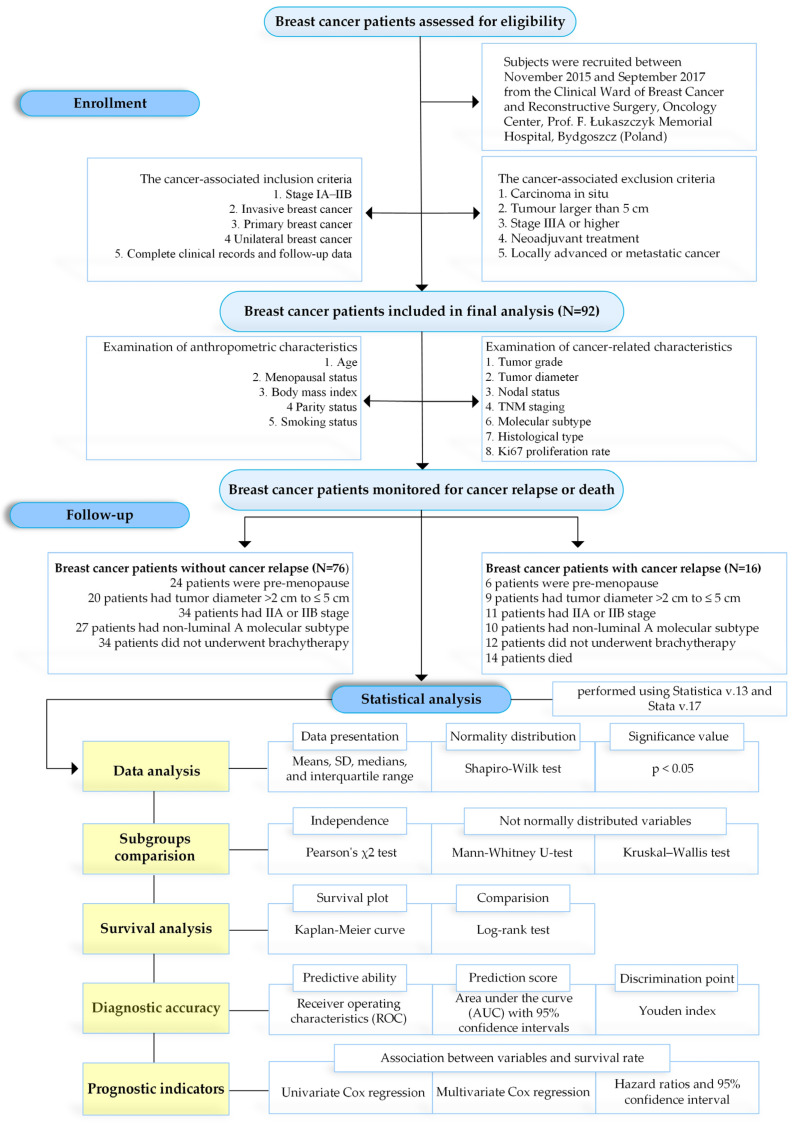
Study design of IBrC patients’ eligibility.

The investigated group included 92 patients diagnosed with IBrC based on pathological examination. The average observation time for survival was 5.83 years. Follow-up information was available in all examined patients (100%). The key elements of the study design including setting, location, periods of recruitment, participant characteristics, and eligibility criteria, which are presented in Figure 1. The survival data details are provided in Table 1.

### 2.2. Ethical Approval

The study was conducted according to the ethical standards of the committee on human experimentation (institutional and national) and according to The Declaration of Helsinki of 1975, as revised in 2000. All eligible study participants provided written informed consent before enrollment (reference number: KB/547/2015).

### 2.3. Tumor Characteristics

The histological type was assessed according to the World Health Organization classification system. The carcinoma samples were also evaluated for histological grade based on Elston–Ellis classification. The individual stage of the tumor at diagnosis was established via the TNM staging system using the American Joint Committee on Cancer (AJCC; 7th edition). The molecular subtype was defined by applying immunohistochemical (IHC) evaluation of hormonal receptors, HER2 and Ki67 mitotic index expressions. Moreover, all the studied patients provided a complete history and underwent clinical examinations.

### 2.4. Therapeutic Procedures

All patients were treated according to the National Comprehensive Cancer Network (NCCN) Guidelines for Practice. In seventy-five subjects, breast-conserving surgery (BCS) was performed, while eight individuals underwent a standard mastectomy, and nine opted for modified radical mastectomy (MRM). All surgery treatment procedures were conducted under standardized conditions. A detailed treatment strategy is included in our previous manuscript [14] and Table 1.

### 2.5. Immunohistochemistry (IHC) Staining Analysis

The detection of ER, PR, HER2, and Ki67 expression in paraffin block of breast cancer tissues was performed using standard immunohistochemistry staining protocols. The ER and PR were assessed according to SCO/CAP guidelines on hormone receptor testing in breast cancer using SP1 and 1E2 primary antibodies (Ventana Medical Systems, Tucson, Arizona, USA), respectively. Tumors were treated as positive for ER and PR if at least 1% of tumor nuclei were stained, irrespective of staining intensity. For the HER2 semi-quantitative detection, the rabbit monoclonal primary antibody VENTANA anti-HER2/neu (4B5) was used with a VENTANA aperture for staining the IHC microscopic slide (Benchmark Ultra, Roche-Ventana). The intensity of the HER2 expression was scored as HER2-negative = 0 or 1+, and HER2-positive = 3+. Tumors with a 2+ score were recognized as equivocal and tested by fluorescence in situ hybridization (FISH) using a dual HER2/Cep17 probe. The Ki67 antigen was assessed as a percentage of nuclei-stained cells of all the cancer cells by applying a monoclonal mouse antibody (Auto-stainer Link 48, Agilent Technologies, Santa Clara, CA, USA). To define cases with high/low proliferation, the Ki67 expression was tested using a 20% cut-off point as the limit.

### 2.6. Statistical Methods

Analysis was performed using Statistica version 13. Data were presented as means, SD, medians, and lower and upper quartiles (Q1–Q3). To assess the normality of the distribution of the variables, the Shapiro–Wilk test was applied. The Pearson’s χ^2^ test was used to determine the independence between categorical variables. Differences between subgroups for not normally distributed data were performed using the Mann–Whitney U-test or the Kruskal–Wallis test. The disease-free survival and overall survival analysis were determined using the Kaplan–Meier estimation and compared using the log-rank test. The Kaplan–Meier plots were created using Stata version 17. Receiver operating characteristic (ROC) curves were used for checking the selected biomarkers’ diagnostic and predictive ability. The area under the curve (AUC) was calculated to estimate the diagnostic accuracy. The optimal threshold values were established based on the Youden criteria. Each investigated parameter was characterized by diagnostic sensitivity and specificity. Univariate and multivariate Cox regression analyses were conducted to estimate the hazard ratios and 95% Cls for potential IBrC recurrence and cancer-specific death prognostic factors. The level of significance value was defined at *p* < 0.05.

## 3. Results

### 3.1. Prospective Cohort (92 Cases)

The study sample consisted of 92 women of Polish descent with a median age of 55 years (Q1–Q3: 49–59 years) and mean body mass index (BMI) of 25.06 kg/m^2^ (Q1–Q3: 22.54 –28.72 kg/m^2^). Of the 92 patients in the IBrC cohort, 30 were pre-menopause and 62 were postmenopausal. Twenty-two (24%) women identified as current smokers. Of those assessed, nine (10%) were nulliparous. The majority of subjects, 65 (70%), had one or two births previously, while 18 (20%) reported three or more previous births. In the studied group with invasive cancers, the histological grade was 1 (low) in 5 (5%), 2 (intermediate) in 69 (75%), and 3 (high) in 18 (20%) cases. Most tumors were <2 cm (63 cases, 68%). The median tumor size was 1.5 cm (Q1–Q3: 1.2–2.1 cm). According to AJCC 7th edition for breast cancer, 47 patients (51%) were in stage I, and 45 (49%) were in stage II. Regarding to breast tumor intrinsic (molecular) classification, 55 cases (60%) had tumors classified as the luminal A subtype, whereas 37 (40%) patients had non-luminal A tumors, including luminal B, non-luminal HER2+, and triple negative.

Adjuvant chemotherapies consisting of anthracycline-containing and non-anthracycline-containing drugs were delivered in 35 and 8 patients, respectively. According to endocrine therapy, 43 (57%) women used tamoxifen, 22 (29%) used aromatase inhibitors (AIs), and 11 (14%) received tamoxifen and aromatase inhibitors as a combination treatment. Eleven HER2-positive women needed adjuvant HER2+-targeted therapy (trastuzumab).

Detailed clinicopathological features and the frequency of therapy application are summarized in Table 1.

### 3.2. Comparison of Progression-Free Patients and Patients with Cancer Recurrence According to Clinicopathological Characteristics

Table 2 compares clinicopathological features between the progression-free group and the group with cancer recurrence. The TNM classification system, including cT category describing tumor size, showed that tumors with a diameter > 2 cm were more frequently observed in women with cancer relapse than in the progression-free group (56% vs. 26%, respectively, *p* = 0.0192). Regarding tumor molecular subtypes, the progression-free group tended to have tumors with luminal-A phenotype (ER + PR + HER2−). In contrast, women with cancer recurrence were positive for other molecular subtypes, including luminal-B HER2-negative, luminal-B HER2-positive, and non-luminal HER2-positive or triple-negative subtypes (63% vs. 37%, respectively; *p* = 0.0455). IBrC clinical outcome was also associated with brachytherapy administration (*p* = 0.0278). Among the 76 progression-free patients, 42 (55%) had received brachytherapy, whereas, among the 16 cases with confirmed cancer recurrence, only 4 (25%) had received that form of adjuvant treatment.

### 3.3. Analysis of Disease-Free Survival and Overall Survival by Demographic and Clinical Characteristics

Table 3 and Table 4 compare disease-free survival and overall survival between groups stratified by demographic and clinical characteristics. The analysis revealed that most of the anthropometric (age, BMI, smoking status, parity status), histopathological (histological type and grading), and clinical (staging, molecular type) parameters had no significant impact on the patients’ overall survival (OS) and disease-free survival (DFS).

It was found that only menopausal status and the value of Ki67 significantly correlated with the duration of DFS and OS. Pre-menopause IBrC patients had statistically significantly worse outcomes and shorter DFS with a median of 59 months (*p* = 0.0244) and shorter OS with a median of 59 months (*p* = 0.0214). A higher Ki67 proliferation index was shown to correlate with a poorer prognosis and early recurrence, with a median DFS of 61 months (*p* = 0.0294), and shorter OS, with a median of 62.5 months (*p* = 0.0316).

### 3.4. Survival Analysis

**Figure 2:**
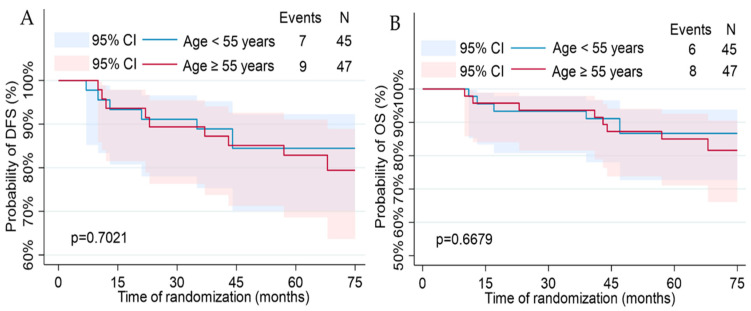

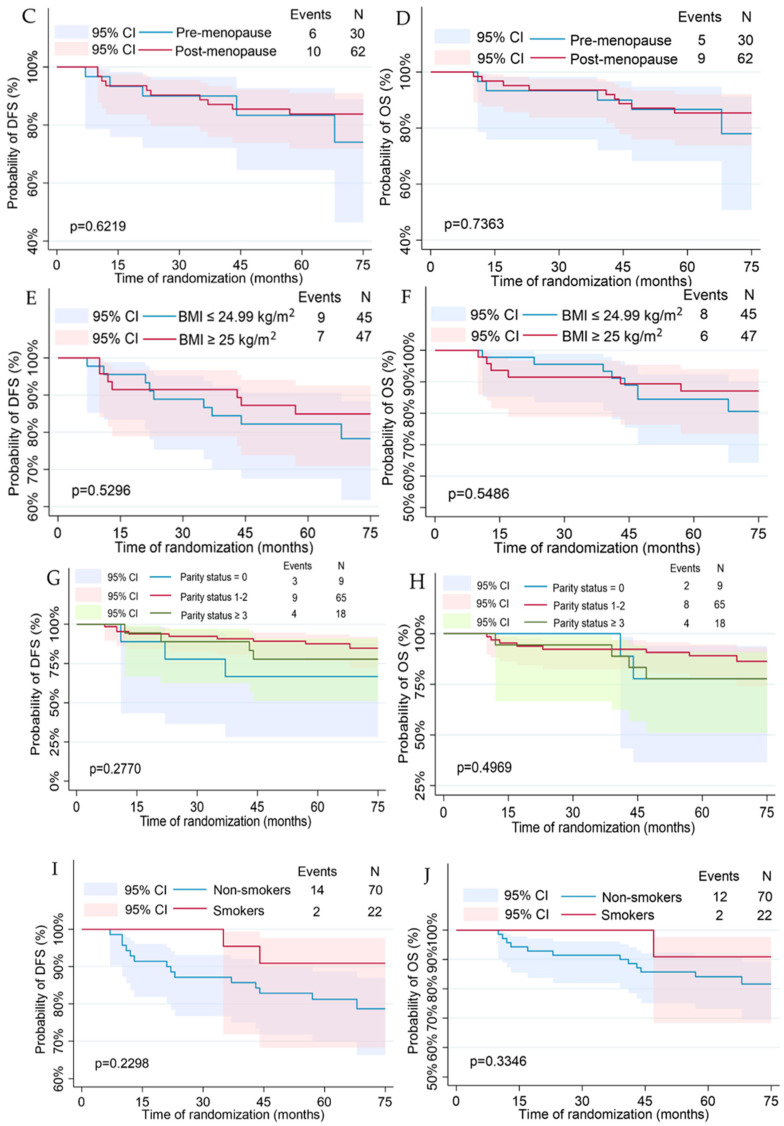
Kaplan–Meier plots for the disease-free and overall survival regarding demographic features: age (**A**,**B**); menopausal status (**C**,**D**); BMI (**E**,**F**); parity status (**G**,**H**); smoking status (**I**,**J**).

**Figure 3:**
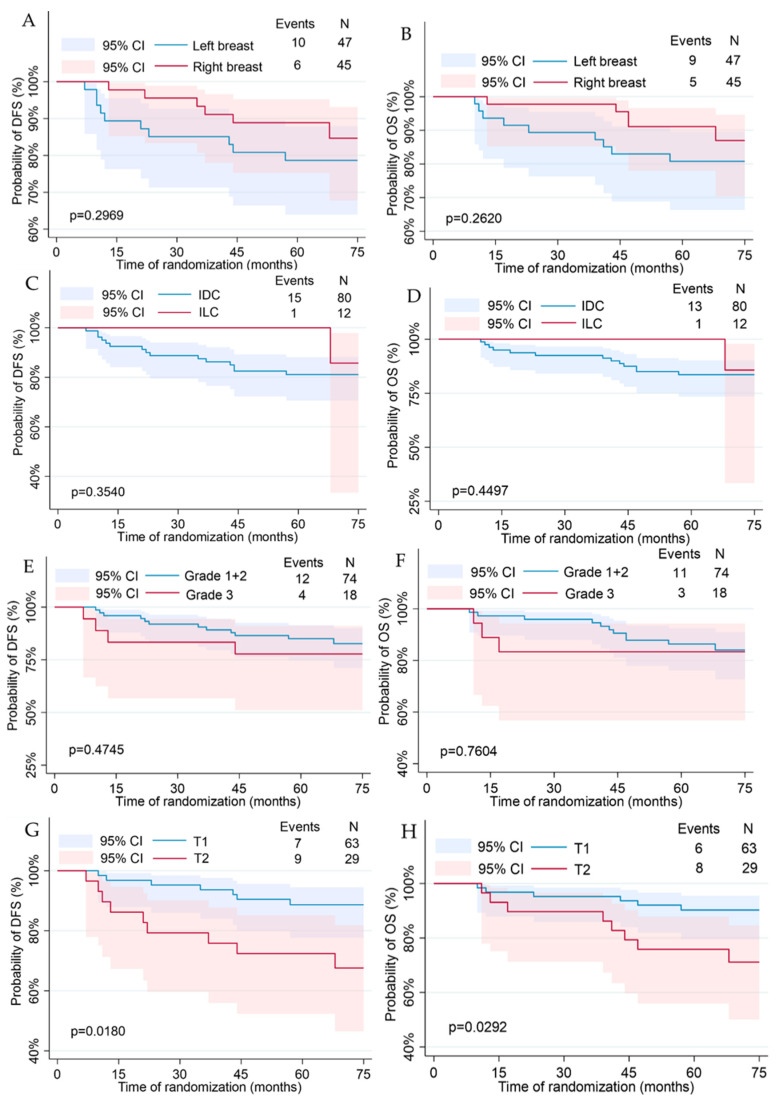

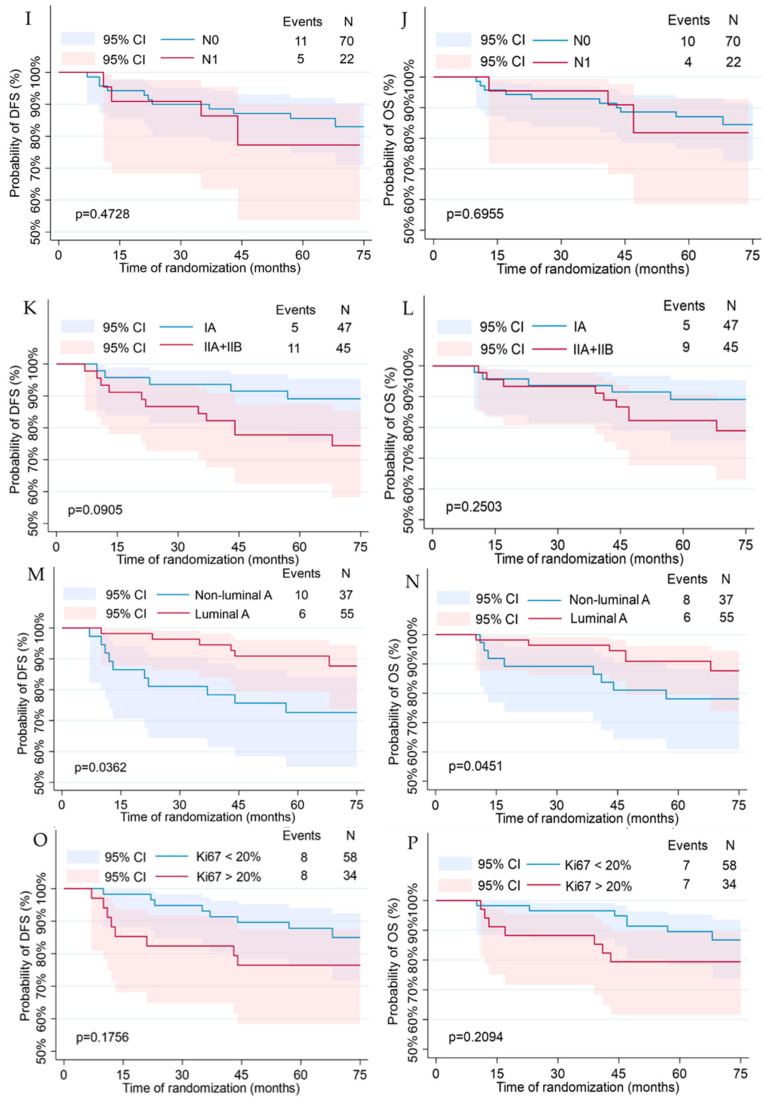
Kaplan–Meier plots for the disease-free and overall survival regarding clinical features: tumor localization (**A**,**B**); histological type (**C**,**D**); Elston–Ellis grading (**E**,**F**); cT category (**G**,**H**); cN category (**I**,**J**); TNM (**K**,**L**); molecular type (**M**,**N**); Ki67 (**O**,**P**).

**Figure 4:**
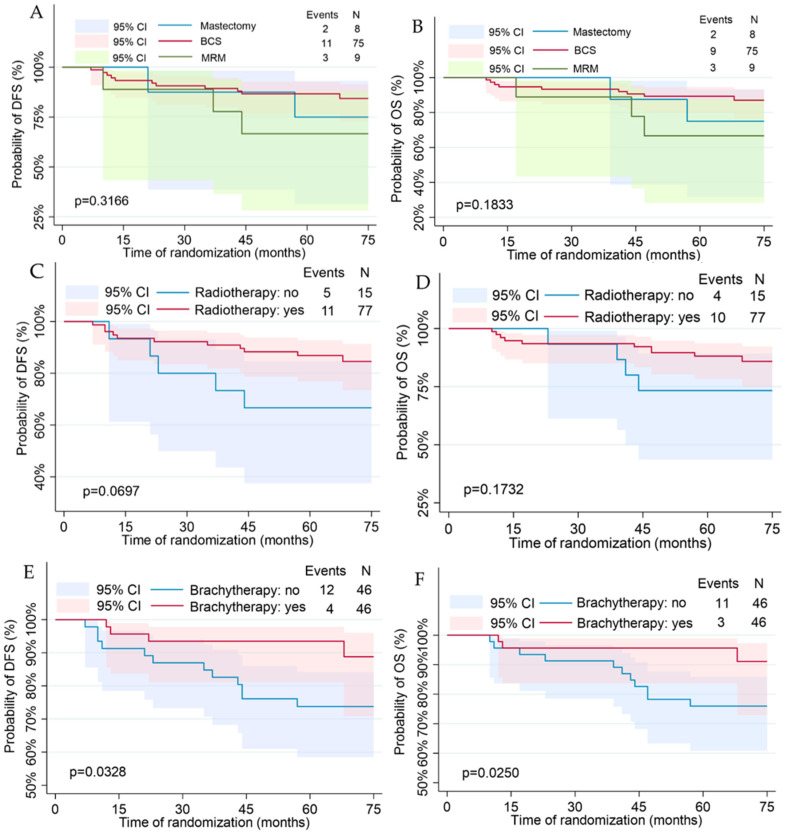

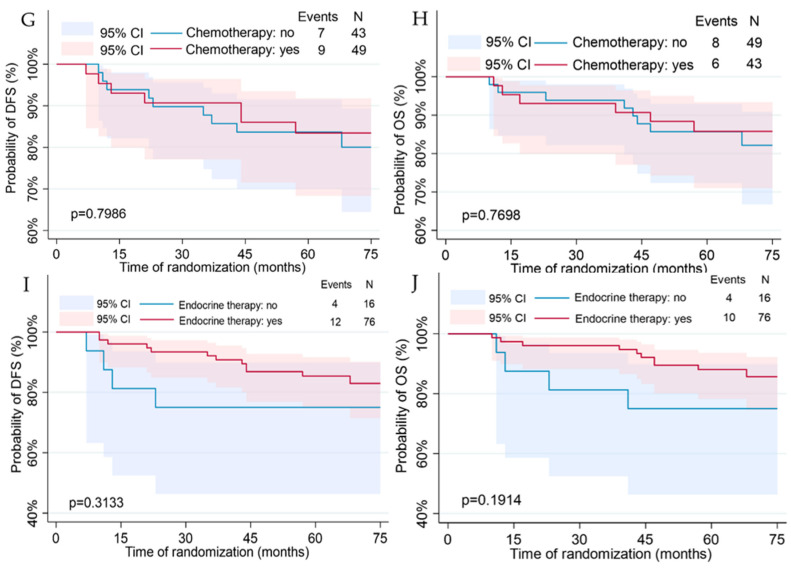
Kaplan–Meier plots for the disease-free and overall survival regarding treatment: surgery type (**A**,**B**); radiotherapy (**C**,**D**); brachytherapy (**E**,**F**); chemotherapy (**G**,**H**); endocrine therapy (**I**,**J**).

Disease-free survival and overall survival calculated analysis using the Kaplan–Meier curves are shown in Figure 2, Figure 3 and Figure 4. Median DFS was 66.5 months (Q1–Q3: 58–73 months) with a recurrence rate of 17.39%. Median OS was 67 months (Q1–Q3: 58–73 months) with a death rate of 15.22%. By comparing using the log-rank test, differences in the disease-free and overall survival functions were observed only in the variables of tumor diameter, molecular subtype, and brachytherapy administration.

Patients with tumor diameter <2 cm (T1 category) were characterized with significantly better DFS and OS rates than subjects with tumor diameter above 2 cm (T2 category) (*p* = 0.0180 for DFS; *p* = 0.0292 for OS), as shown in Figure 3G,H. The estimated 5 year probability of DFS and OS rates in women with tumor diameter <2 cm were 89% and 90%, respectively. In patients with tumor diameter > 2 cm, the estimated 5 year probability of DFS was 73% and OS was 76%.

Based on Kaplan–Meier analysis for molecular subtype, the survival rate was poorer in patients with a type other than luminal A (*p* = 0.0362 for DFS; *p* = 0.0415 for OS). The estimated 5 year probability of DFS and OS rates in patients with luminal A was 91% for both outcomes. In patients with molecular types other than luminal A, the estimated 5 year probability of DFS was 73% and OS was 78% (Figure 3M,N).

Follow-up using Kaplan–Meier analysis revealed a significantly better and longer disease-free (*p* = 0.0328) and overall survival outcome (*p* = 0.0250) in women who received brachytherapy (Figure 4E,F). Compared with patients with a lack of brachytherapy, applying brachytherapy was correlated with a decreased 5 year incidence of IBrC recurrence (6% vs. 62.5%).

### 3.5. The ROC Analysis for Diagnostic Accuracy of Potential Prognostic Factors

The ROC analysis was designed to estimate the diagnostic accuracies of the studied basic clinicopathological parameters for predicting the risk of IBrC recurrence (Table 5). The AUC specified 95% confidence intervals. Among the individual factors, a tumor diameter yielded the most significant AUC (AUC = 0.7130; *p* = 0.0014). Based on the Youden index, the tumor diameter of 1.6 cm with a specificity of 60.5% and a sensitivity of 75.0% was determined as the best threshold value to differentiate patients with cancer recurrence from subjects without cancer relapse. These results showed that tumor diameter was considered as the key predictive indicator of disease relapse. For other studied clinicopathological factors, the solid diagnostic potential for predicting the progression of the disease was not reached (*p* > 0.05).

### 3.6. Evaluation of Potential Predictive Indicators Based on Cox Regression Models

Potential influential factors, including differences between the two groups and factors related to clinical features, were screened to find the possible indicators for the patient group. Univariate and multivariate Cox regression models assessing the relationship of selected potentially prognostic variables with DFS and OS are presented in Table 6 and Table 7. Variables with a significant *p*-value in the univariate survival model were considered further in the multivariate model. According to the results of univariate analysis, molecular subtype (*p* = 0.0454) and brachytherapy (*p* = 0.0438) were significantly associated with disease-free survival and tumor diameter (*p* = 0.0385), and brachytherapy (*p* = 0.0377) was related to OS.

In a multivariate COX analysis, applying other relevant variables into the model, we noted a significant relationship between molecular subtype, brachytherapy administration, and tumor diameter with the risk of IBrC progression (*p* < 0.05). The regression analysis revealed that luminal A IBrC and brachytherapy administration was positively associated with a better and prolonged DFS (HR = 3.09, 95% CI = 1.12–8.53, *p* = 0.0293; HR = 3.51, 95% Cl = 1.13–10.91, *p* = 0.0302, respectively). Similarly, tumor size < 2 cm (T1) and brachytherapy administration were positively correlated with better outcomes and longer overall survival (HR = 2.89, 95% CI = 1.00–8.53, *p* = 0.0498; HR = 3.68, 95% Cl = 1.03–13.23, *p* = 0.0457), respectively) According to these results, tumor diameter, a molecular subtype of breast cancer, and brachytherapy administration were identified as independent predictor factors of disease-free and overall survival.

## 4. Discussion

Given the increasing focus on molecular prognostic tests in recent times, there is a tendency to disregard the crucial role played by traditional clinical and pathological factors in predicting breast cancer prognosis [15]. In the era of advanced molecular diagnostic tests, the traditional factors still remain essential in determining prognosis and guiding therapeutic decisions for individuals facing a new diagnosis of invasive breast cancer. This study investigated the prognostic role of basic clinical parameters used in diagnosing breast cancer as potential prognostic factors of cancer recurrence and overall survival. We found that molecular classification, tumor diameter, Ki67 expression, and brachytherapy administration may be the most potent predictors of cancer recurrence and overall survival.

### 4.1. Tumor Diameter as a Prognostic Indicator of Survival

Findings from this study showed that tumor diameter has a significant relationship with disease relapse and overall survival in IBrC patients. Regarding our analysis, patients with tumor diameter > 2 cm to ≤5 cm (T2) demonstrated poorer clinical outcomes compared to patients with tumor diameter < 2 cm (T1). Tumor size > 2 cm—a cut-off point between the T1 and T2 group—gave a reliable estimate of disease-free and overall survival. Surprisingly, regarding ROC analysis, we found the optimal cut-off point of tumor diameter to be 1.6 cm, discriminating between cancer-relapse and non-relapse disease patients. Presumably, this preliminary observation suggests changing the direction of breast cancer therapy. Perhaps, shifting the cut-off point from 2 cm to lower values will reduce the incidence of recurrence in the IBrC patients. This innovative approach may encourage clinicians to consider introducing more determined surgical procedures than breast-conserving surgery or removing a wider margin of healthy tissue in patients with a smaller tumor diameter, which will reduce the mortality of patients with early stage IBrC. Our results are consistent with the observations of Neves Rebello Alves et al. and Gu et al. [15,16].

Based on Kaplan–Meier curves with log-rank, patients with tumor size < 2 cm displayed significantly more favorable prognosis (longer DFS and OS) than those with tumor diameter > 2 cm to ≤ 5 cm. By applying a multivariate Cox model, we verified the prognostic value of the tumor size as an independent indicator of overall survival. Women in the group with T2 presented nearly a threefold increased risk of death due to systemic metastatic disease (HR = 2.89; 95% Cl = 1.00–8.35). This negative association between tumor size and survival is perhaps not surprising, especially considering the cancer pathogenesis and tumor invasiveness, as the likelihood of metastasis development increases with tumor growth, irrespective of the number of lymph nodes [17,18,19]. Nevertheless, this is also evident in our study. Our findings corroborate the research performed by Mahmood et al., which noted the strong impact of tumor diameter on survival and reported that more patients with tumor diameter ≤ 2 cm presented an overall survival of more than ten years [20]. Furthermore, Shi et al. noted that lymph node metastasis, tumor diameter, and Ki67 expression were risk factors affecting the 5 year DFS rates in patients with ultra-low HER2 expression [21]. The above-mentioned studies emphasize the importance of commonly used clinicopathological biomarkers as still valid and relevant prognostic factors.

### 4.2. Molecular Classification as a Prognostic Indicator of Survival

The relationship between IBrC molecular subtype and survival has been well understood for many years and has also been confirmed by our results. Our analyses revealed that most patients with cancer progression displayed tumors in molecular subtypes other than luminal-A (10 vs. 6 women in the chi-squared test), so we suggest that patients with the luminal A subtype have the most favorable survival prognosis. Based on Kaplan–Meier survival curves, we found that intrinsic subtypes are a single biomarker of DFS and OS duration in IBrC patients. The estimated 5 year probability of DFS and OS rates in patients with luminal-A cancer subtype was 91%, but in patients with non-luminal-A cancer, the estimated 5 year probability of DFS was 74% and OS was 79%. A positive association between the luminal-A tumors and survival was definitely confirmed using the Cox regression model, which revealed that a tumor with a molecular cancer subtype other than luminal-A is associated with a 3.09 times higher risk of disease recurrence. In a fascinating study aimed at identifying breast cancer molecular phenotypes as a predictor of survival, Pracella et al. reported that ER-positive cancers have the best outcome. At the same time, triple-negative and HER2-positive non-luminal types showed the shortest and the poorest survival rates. Using the Cox proportional hazard model, the authors also confirmed an independent and positive influence on breast cancer survival for luminal-A tumors [22]. Based on the available evidence, luminal-A tumors are typically low grade with a highly favorable prognosis and low expression of proliferation-related genes. Additionally, this subtype strongly corresponds to adjuvant endocrine therapy targeting estrogen receptors (such as tamoxifen and aromatase inhibitors) and, as a result, tends to grow at a slower rate and occurs to be usually non-metastasising when compared with the other molecular subtypes [7,23].

### 4.3. Ki67 Proliferation Index as a Prognostic Indicator of Survival

Numerous researchers have investigated a relationship between Ki67 value and the probability of IBrC recurrence, which is also evident in the present study. Our results strongly demonstrate that the relapse rate and cancer-specific deaths highly depend on the expression of Ki67. In IBrC patients with Ki67 staining < 20%, we observed longer disease-free survival and overall survival as well, compared to IBrC patients with Ki67 staining > 20% (68.5 vs. 61 months for median DFS and 70 vs. 62.5 months for median OS), what brightly suggest that Ki67 expression > 20% contributes to a higher risk of cancer recurrence and specific death. Unfortunately, we found no significant correlation between Ki67 value and the probability of DFS and OS in Kaplan–Meier and Cox regression analyses. A systematic review and meta-analysis by Stuart-Harris et al. involving 32,825 patients demonstrated a strong association between Ki67 value and future clinical outcomes. In particular, the authors reported that the Ki67 positivity value is associated with a higher risk of recurrence and also acts as an independent prognostic factor predicting the incidence of lymph node metastasis [24]. These findings corroborate the results of Rakha et al.’s study showing that high Ki67 expression is a prognostic factor for overall and disease-free survival and may be successfully used in clinical routine [25]. Currently, many available diagnostic markers evaluate how rapidly the tumor is growing. For many years, the nuclear protein Ki67 has been strongly associated with cellular proliferation [26,27]. Because Ki67 remains active during the G1, S, G2, and M phases of the cell cycle, this protein acts as a great marker of cell proliferation, indirectly reflecting the oncogenesis status [28]. Thus, Ki67 is commonly used in a clinical routine as a proliferative biomarker and an essential diagnosis tool [29,30].

### 4.4. Brachytherapy Application as a Prognostic Indicator of Survival

The culmination of our study was an evaluation of the effect of the applied treatment on disease-free and overall survival duration. We observed that the incidence of cancer recurrence was significantly higher in women who did not receive brachytherapy after whole-breast irradiation than in women who underwent brachytherapy (reoccurrence rate 75% vs. 25% in the chi-squared test). Furthermore, our analysis, including Kaplan–Meier survival analysis, reported a more favorable prognosis according to 5 year disease-free survival and overall survival for patients with an extra irradiation dose to the tumor bed. The calculated probability of 5 year DFS and OS from the Kaplan-Meier curve was 94% and 96% for patients who underwent brachytherapy and 73% and 76% for patients who did not, respectively. Finally, the rate risk calculated from HR for DFS and OS showed a highly significant association, indicating that receiving brachytherapy may predict both cancer recurrence and cancer-specific death. Based on these results, we noted that patients who did not receive brachytherapy had a 3.59 times higher risk of disease recurrence and a 3.68 times higher risk of death from cancer than those who underwent brachytherapy.

For breast cancer, it is well-established that vicinity of the tumor bed is a potential source of ipsilateral local recurrence and cancer relapse. Thus, it seems equitable to consider a supplemental dose of radiation given to the tumor bed after whole-breast irradiation [31]. Interestingly, after 20 years of follow-up, Bartelink et al. noted no significant improvement in long-term overall survival but found the measurable benefit of a local radiation boost after whole-breast irradiation regarding local control rates. The mentioned study proves that a boost dose did reduce the incidence of ipsilateral breast tumor recurrence and emphasizes the significant role of dose escalation to the tumor bed [32]. A similar observation was received by Kauer-Dorner et al. who summarized the results of several randomized brachytherapy studies worldwide and demonstrated an evident benefit of brachytherapy boosting in order to minimize the local recurrence rate [33]. Another study, “boost vs. no boost”, conducted by Polgár et al., revealed an evident benefit from administrating brachytherapy, summarizing results of three randomized trials that confirm that an extra-dose brachytherapy boost after whole-breast irradiation significantly decreased the local recurrence rate. The authors of the mentioned research demonstrated that local brachytherapy boost significantly minimizes 5 year local recurrence rates from 7.3–13.3% in the non-boost groups to 4.3–6.3% in the boost groups [34]. Although brachytherapy is not a standard cancer treatment option according to NCCN guidelines, given the multifaceted nature of brachytherapy, a systematic exploration into its benefits holds significant promise for contributing novel insights to the existing body of scientific literature. Numerous studies have demonstrated that brachytherapy yields comparable oncologic outcomes to conventional external beam radiation therapy (EBRT) in terms of local control, disease recurrence rates, and overall survival. Results from available prospective clinical trials have confirmed the efficacy of brachytherapy as a viable alternative to EBRT for selected patients with early stage breast cancer, further supporting its clinical utility. Additionally, compared with EBRT, brachytherapy has undoubted advantages in terms of shortening the total treatment time from 5–7 weeks to 4–5 days, offering greater convenience for patients. Moreover, it is anticipated to decrease the incidence of complications such as radiation-induced reactions, telangiectasia, and fibrosis. Brachytherapy is also associated with better cosmetic outcomes compared to traditional whole-breast radiation therapy. By focusing radiation directly on the tumor bed, brachytherapy helps preserve the appearance of the breast, minimizing changes in shape, texture, and pigmentation. Thus, brachytherapy represents a valuable therapeutic modality in the comprehensive management of breast cancer, offering advantages in targeted radiation delivery, cosmetic preservation, treatment efficiency, oncologic efficacy, clinical adaptability, and cost-effectiveness. As ongoing research continues to refine treatment protocols and expand our understanding of brachytherapy’s role in breast cancer care, its benefits are poised to further enhance patient outcomes and quality of life in the years ahead. This pattern of results is consistent with our investigation, so we suggest that these findings may encourage surgeons and clinicians to consciously decide to administrate extra-dose brachytherapy boost to patients with IBrC to reduce the risk of a local relapse within the treated breast. Undoubtedly, the oncology community is eagerly awaiting the moment when this method will become the standard of care for patients with early breast cancer [33,35,36].

### 4.5. Advantage of Traditional Prognostic Biomarkers over Molecular Polygenic Prognostic Tests

The constantly increasing number of breast cancer cases indicates that there is an absolute need to better understand the cellular and molecular basis of this cancer to improve and increase the effectiveness of prevention and therapy. Globally, the percentage of 5 year survival due to breast cancer is still alarming, amounting to approximately 96% for early stage breast cancer patients, whereas for newly diagnosed metastatic cases, it is around 38% [37]. Therefore, it appears reasonable to examine factors established as significant in predicting the 5 year survival of breast cancer (BC) patients and consider their relevance in survival outcomes 10 years post-diagnosis and beyond. Nowadays, available markers include both well-established traditional clinical factors and biochemical-molecular factors. In the era of modern medical practice, which places significant emphasis on molecular multigene assays, e.g., Oncotype DX or MammaPrint, the traditional prognostic markers in breast cancer are often overlooked, despite their paramount importance. However, through our research, we aim to revisit the foundational aspects and underscore the significance of traditional biomarkers in predicting survival in IBrC patients. Although advanced molecular markers offer additional insights into the biological characteristics of breast tumors, traditional biomarkers continue to play a central role in breast cancer diagnosis and management due to their widespread availability, cost-effectiveness, proven clinical utility, and standardization. Thus, the basic breast cancer biomarkers are still more sufficient to guide IBrC patients than multigene signatures. Compared to molecular prognostic tests, traditional biomarkers are routinely assessed in clinical practice and are widely available in most healthcare settings. They are typically included in standard pathology reports, making them easily accessible to healthcare providers. Moreover, they often require standard immunohistochemical or fluorescent in situ hybridization techniques, which are well-established and cost-effective. The use of these factors also provides valuable information for longitudinal monitoring of disease progression and treatment response. Interestingly, in most breast cancer types, the occurrence of lymph node metastasis can be predicted by tumor size. Changes in tumor size, hormone receptor status, or HER2 expression over time can guide adjustments in treatment strategies. Furthermore, routine biomarkers have been extensively studied and validated over many years, establishing their clinical utility in breast cancer diagnosis, prognosis, and treatment decision making. They have well-defined cutoff points and guidelines for interpretation. Last but not least, basic breast cancer biomarkers, such as hormone receptor status and HER2 expression, have proven predictive value for response to specific targeted therapies, such as hormone therapy or HER2-targeted agents. Hence, traditional clinicopathological features used in breast cancer diagnosis serve as important pillars of clinical decision making in breast cancer care, which cannot be forgotten while focusing on molecular prognostic tests in recent years [15,38,39,40].

### 4.6. Strengths and Limitations of the Study

Our study has some limitations, the first being the limited sample size and lack of a control group. It would be interesting to evaluate the same results in large trials. Hence, the findings of this study may be subject to bias, warranting validation through larger-scale studies to corroborate our results. Moreover, we enrolled patients at an early stage of breast cancer, excluding those with metastases, thus limiting our ability to assess the prognostic significance for larger and more advanced tumors. Lastly, the study’s retrospective design may hinder the collection of comprehensive data on relevant confounders or prognostic factors, such as patient comorbidities, lifestyle factors, or socioeconomic status. Failure to account for these variables adequately could confound the observed associations and limit the study’s ability to draw robust conclusions about the prognostic value of brachytherapy, molecular subtype, and tumor diameter in breast cancer. Nevertheless, this study’s main conclusions are consistent with other authors’ recent trial estimates. The strengths of this study include a broad-parameter subgroup analysis indicating the potential influence of various factors on the DFS and OS duration and an extensive analysis of the predictive value of numerous factors as an independent risk predictor of cancer recurrence and cancer-specific death in women with IBrC. However, further studies on larger populations including the control group are necessary; therefore, the obtained results should be interpreted with caution.

## 5. Conclusions

Our results suggest that disease-free survival and overall survival may depend on the tumor diameter, molecular classification, expression of Ki67 proliferation marker, and brachytherapy administration. This study among patients with IBrC may provide more insight into the impact of traditional clinicopathological factors on their future outcomes. While essential recurrence and cancer-specific death risk factors for IBrC include age, menopausal status, nodal involvement, histological grade, and type of received therapy, this analysis presents that the tumor size < 2 cm, Ki67 expression < 20%, luminal-A molecular subtype, and use of effective extra-dose brachytherapy boost could most probably appear as having the most significant positive impact on IBrC disease-free and overall survival and presumably may be considered as independent prognostic factors of breast cancer.

## Figures and Tables

**Table 1:** Histo-patho-therapeutical profile of study participants.

Histo-Patho-Therapeutical Data	Number of Patients (%)
Tumor site	
Left	47 (51%)
Right	45 (49%)
Tumor grade	
Low/1 + Intermediate/2	74 (80%)
High/3	18 (20%)
Tumor diameter	
≤2 cm (T1)	63 (68%)
>2 cm to ≤5 cm (T2)	29 (32%)
Nodal status	
No regional lymph nodes metastasis	70 (76%)
Axillary lymph node metastasis	22 (24%)
TNM staging	
IA	47 (51%)
IIA + IIB	45 (49%)
Hormone receptor status	
ER-detected	80 (87%)
ER-undetected	12 (13%)
PR-detected	74 (80%)
PR-undetected	18 (20%)
HER2-detected	11 (12%)
HER2-undetected	81 (88%)
Intrinsic subtype	
Luminal A	55 (60%)
Other	37 (40%)
Histological type	
Invasive ductal carcinoma	80 (87%)
Invasive lobular carcinoma	12 (13%)
Ki67 proliferation rate	
<20%	58 (63%)
>20%	34 (37%)
Type of surgery	
Mastectomy	8 (9%)
Modified radical mastectomy	9 (10%)
Breast-conserving surgery	75 (81%)
Irradiation	
+	77 (84%)
−	15 (16%)
Brachytherapy	
+	46 (50%)
−	46 (50%)
Chemotherapy	
+	43 (47%)
−	49 (53%)
Endocrine therapy	
+	76 (83%)
−	16 (17%)
HER2+-targeted therapy	
+	11 (12%)
−	81 (88%)
Disease relapse	
Yes	16 (17%)
No	76 (83%)
Disease-free survival (months)	
Median (Q1–Q3)	66.5 (58–73)
Died during follow-up	
Yes	14 (15%)
No	78 (85%)
Overall survival (months)	
Median (Q1–Q3)	67 (58–73)

ER: estrogen receptor; PR: progesterone receptor; HER2: human epidermal growth factor receptor 2; Q1–Q3: lower and upper quartile. Other intrinsic subtypes included luminal B, non-luminal HER2+, and triple-negative tumors.

**Table 2:** Comparison of progression-free patients and patients with cancer recurrence.

Histo-Patho-Therapeutical Data	Patients Without Progression (*n* = 76)	Patients with Progression (*n* = 16)	*p* *
	*n* (%)		
Tumor site Left Right	37 (49%) 39 (51%)	10 (62%) 6 (38%)	0.3150
Tumor grade Low/1 + Intermediate/2 High/3	62 (82%) 14 (18%)	12 (75%) 4 (25%)	0.5466
Tumor diameter ≤2 cm (T1) >2 cm to ≤5 cm (T2)	56 (74%) 20 (26%)	7 (44%) 9 (56%)	0.0192
Nodal status No regional lymph nodes metastasis Axillary lymph node metastasis	59 (78%) 17 (22%)	11 (69%) 5 (31%)	0.4491
TNM staging IA IIA + IIB	42 (55%) 34 (45%)	5 (31%) 11 (69%)	0.0807
Hormone receptor status ER-detected ER-undetected PR-detected PR-undetected HER2-detected HER2-undetected	66 (87%) 10 (13%) 62 (82%) 14 (18%) 9 (12%) 67 (88%)	14 (87%) 2 (13%) 12 (75%) 4 (25%) 2 (13%) 14 (87%)	0.9434 0.5466 0.9412
Intrinsic subtype Luminal A Other	49 (64%) 27 (36%)	6 (37%) 10 (63%)	0.0455
Histological type Invasive ductal carcinoma Invasive lobular carcinoma	65 (86%) 11 (14%)	15 (94%) 1 (6%)	0.0969
Ki67 proliferation rate <20% >20%	50 (66%) 26 (34%)	8 (50%) 8 (50%)	0.2343
Type of surgery Mastectomy Modified radical mastectomy Breast-conserving surgery	6 (8%) 6 (8%) 64 (84%)	2 (13%) 3 (18%) 11 (69%)	0.3163
Irradiation + −	66 (87%) 10 (13%)	11 (69%) 5 (31%)	0.0750
Brachytherapy + −	42 (55%) 34 (45%)	4 (25%) 12 (75%)	0.0278
Chemotherapy + −	36 (47%) 40 (53%)	7 (44%) 9 (56%)	0.7920
Endocrine therapy + −	64 (84%) 12 (16%)	12 (75%) 4 (25%)	0.3770
HER2+-targeted therapy + −	9 (12%) 67 (88%)	2 (13%) 14 (87%)	0.9412

ER: estrogen receptor; PR: progesterone receptor; HER2: human epidermal growth factor receptor 2. Other intrinsic subtypes included luminal B, non-luminal HER2+, and triple-negative tumors. *p* * for baseline differences between subjects without progression and subjects with progression in the chi-squared test; underlined *p*-values denote significant differences.

**Table 3:** Impact of anthropometric and demographic variables on disease-free and overall survival.

	DFS (Months)		OS (Months)			
	Mean ± SD	Median (Q1–Q3)	Mean ± SD	Median (Q1–Q3)	*p* *	*p* **
Age Age < 55 years Age ≥ 55 years	59.36 ± 17.60 61.47 ± 18.25	63.00 (56.00–71.00) 68.00 (59.00–74.00)	60.87 ± 15.63 63.32 ± 15.67	65.00 (58.00–71.00) 69.00 (59.00–74.00)	0.2087	0.1792
Climacteric stage Pre-menopause Post-menopause	57.50 ± 17.10 61.85 ± 18.20	59.00 (56.00–70.00) 70.00 (59.00–74.00)	59.13 ± 15.40 63.56 ± 15.63	59.00 (56.00–70.00) 70.00 (59.00–74.00)	0.0244	0.0214
Quetelet index (BMI) ≤24.99 kg/m^2^ ≥25 kg/m^2^	59.60 ± 18.41 61.23 ± 17.50	66,00 (56.00–73.00 67.00 (58.00–73.00)	62.36 ± 14.33 61.89 ± 16.90	67.00 (58.00–73.00) 67.00 (58.00–73.00)	0.7900	0.8941
Parity status 0 1–2 3 and more	54.33 ± 25.04 61.74 ± 16.66 58.78 ± 18.43	71.00 (37.00- 73.00) 67.00 (59.00–73.00) 63.00 (56.00–74.00)	64.00 ± 13.92 62.46 ± 15.75 59.95 ± 16.52	72.00 (53.00–73.00) 67.00 (59.00–73.00) 63.00 (56.00–74.00)	0.8880	0.7250
Smoking status Yes No	65.82 ± 10.31 58.74 ± 19.41	68.50 (63.00–74.00) 65.00 (56.00–73.00)	66.50 ± 8.47 60.74 ± 17.08	68.50 (63.00–74.00) 67.00 (57.00–73.00)	0.1940	0.2727

DFS: disease-free survival; OS: overall survival; Q1–Q3: lower and upper quartile; BMI: body mass index. In the Mann–Whitney U test/Kruskal–Wallis test: * *p* for baseline differences between subgroups for DFS analysis; ** *p* for baseline differences between subgroups for OS analysis; underlined *p*-values denote significant differences.

**Table 4:** Impact of histo-patho-therapeutical variables on disease-free and overall survival.

	DFS (Months)		OS (Months)			
	Mean ± SD	Median (Q1–Q3)	Mean ± SD	Median (Q1–Q3)	*p* *	*p* **
Tumor grade Low/1 + Intermediate/2 High/3	61.76 ± 16.41 55.00 ± 22.67	67.00 (58.00–73.00) 63.50 (49.00–71.00)	63.38 ± 13.76 56.95 ± 21.37	67.50 (58.00–73.00) 66.00 (49.00–71.00)	0.2318	0.3154
Tumor diameter ≤2 cm (T1) >2 cm to ≤5 cm (T2)	62.54 ± 14.21 55.86 ± 23.65	65.00 (58.00–73.00) 68.00 (44.00–72.00)	63.11 ± 13.72 59.97 ± 19.19	67.00 (58.00–73.00) 70.00 (58.00–72.00)	0.7139	0.9731
Nodal status No regional lymph nodes metastasis Axillary lymph node metastasis	60.81 ± 17.69 59.23 ± 18.80	66.00 (58.00–73.00) 66.50 (53.00–71.00)	62.04 ± 15.99 62.36 ± 14.71	67.00 (58.00–73.00) 67.50 (56.00–71.00)	0.5631	0.6762
TNM staging IA IIA + IIB	62.02 ± 15.10 58.78 ± 20.41	65.00 (58.00–73.00) 68.00 (56.00–72.00)	62.02 ± 15.10 62.22 ± 16.30	65.00 (58.00–73.00) 70.00 (59.00–72.00)	0.8295	0.8051
Intrinsic subtype Luminal A Other	64.09 ± 13.19 55.00 ± 22.26	68.00 (59.00–73.00) 63.00 (49.00–72.00)	64.36 ± 12.72 58.78 ± 18.83	68.00 (59.00–73.00) 64.00 (56.00–72.00)	0.0756	0.2014
Histological type Invasive ductal carcinoma Invasive lobular carcinoma	59.21 ± 18.75 68.58 ± 5.70	65.00 (56.00–73.00) 68.50 (65.50–73.50)	61.15 ± 16.40 68.58 ± 5.70	66.00 (57.50–73.00) 68.50 (65.50–73.50)	0.1383	0.1812
Ki67 proliferation rate <20% >20%	63.90 ± 14.20 54.53 ± 21.80	69.50 (59.00–73.00) 61.00 (49.00–72.00)	65.14 ± 12.37 56.97 ± 19.09	70.00 (59.00–73.00) 62.50 (49.00–72.00)	0.0294	0.0316
Type of surgery Mastectomy Modified radical mastectomy Breast-conserving surgery	62.38 ± 18.12 54.67 ± 20.72 60.92 ± 17.62	69.50 (58.00–74.00) 63.00 (44.00–65.00) 67.00 (58.00–73.00)	64.63 ± 12.49 56.56 ± 17.93 62.52 ± 15.67	69.50 (58.00–74.00) 63.00 (47.00–65.00) 68.00 (58.00–73.00)	0.4671	0.4186
Irradiation + −	61.56 ± 16.92 54.67 ± 21.87	67.00 (58.00–73.00) 63.00 (37.00–73.00)	62.55 ± 15.62 59.93 ± 15.90	68.00 (58.00–73.00) 64.00 (44.00–73.00)	0.4454	0.6146
Brachytherapy + −	62.50 ± 14.42 58.37 ± 20.72	65.50 (59.00–73.00) 68.50 (49.00–73.00)	63.59 ± 13.13 60.62 ± 17.78	66.50 (59.00–73.00) 69.00 (50.00–73.00)	0.9719	0.9597
Chemotherapy + −	60.65 ± 17.75 60.24 ± 18.16	66.00 (57.00–73.00) 67.00 (58.00–73.00)	61.95 ± 15.94 62.27 ± 15.48	67.00 (58.00–73.00) 67.00 (58.00–73.00)	0.8017	0.7566
Endocrine therapy + −	61.64 ± 15.71 54.69 ± 25.72	66.50 (58.00–73.00) 67.50 (36.00–73.50)	63.24 ± 13.60 56.81 ± 22.75	67.00 (59.00–73.00) 67.50 (45.00–73.50)	0.8323	0.7566
HER2+-targeted therapy + −	65.45 ± 11.06 59.75 ± 18.55	70.00 (57.00–74.00) 65.00 (58.00–73.00)	67.64 ± 8.46 61.37 ± 16.23	70.00 (62.00–74.00) 66.00 (58.00–73.00)	0.3980	0.2468

DFS: disease-free survival; OS: overall survival; Q1–Q3: lower and upper quartile. Other intrinsic subtypes included luminal B, non-luminal HER2+, and triple-negative tumors. In the Mann–Whitney U test/Kruskal–Wallis test: * *p* for baseline differences between subgroups for DFS analysis; ** *p* for baseline differences between subgroups for OS analysis; underlined *p*-values denote significant differences.

**Table 5:** Results of diagnostic accuracy for individual clinicopathological factors.

ROC Data	Age (Years)	BMI (kg/m^2^)	Parity Status	Tumor Size (cm)	Ki67 Expression (%)
AUC	0.523	0.434	0.493	0.713	0.628
Youden index	0.14	0.06	0.11	0.36	0.24
Cut-off threshold	49.00	21.68	0.00	1.60	30.00
Diagnostic sensitivity (%)	87.50	87.50	18.80	75.00	50.00
Diagnostic specificity (%)	26.30	18.40	92.10	60.50	73.70
PPV (%)	20.00	18.40	33.30	28.60	28.60
NPV (%)	90.90	87.50	84.30	92.00	87.50
Diagnostic accuracy (%)	37.00	30.40	79.30	63.00	69.60
*p*-value	0.7609	0.3901	0.9355	0.0014	0.0937

PPV: positive predictive value; NPV: negative prognostic value; BMI: body mass index. Underlined *p*-values denote significant differences.

**Table 6:** Univariate Cox regression model assessing the potential predictive indicators.

Univariate				
Variables	Disease-Free Survival		Overall Survival	
	Hazard Ratio (HR) (95% CI)	*p*-value	Hazard Ratio (HR) (95% CI)	*p*-Value
Age <55 years ≥55 years	1.21 (0.45–3.26)	0.7029	1.26 (0.44–3.63)	0.6689
Climacterium stage Pre-menopause Post-menopause	0.78 (0.28–2.14)	0.6234	0.83 (0.28–2.48)	0.7369
Quetelet index (BMI) ≤24.99 kg/m^2^ ≥25 kg/m^2^	0.73 (0.27–1.96)	0.5319	0.72 (0.25–2.09)	0.5507
Parity status 0 1–2 3 and more	0.37 (0.10–1.38) 0.62 (0.14–2.78)	0.1387 0.9746	1.78 (0.37–8.50) 2.53 (0.45–14.30)	0.8444 0.3323
Smoking status Yes No	0.42 (0.09–1.83)	0.2454	0.49 (0.11–2.17)	0.3454
Tumor site Left Right	1.70 (0.62–4.68)	0.3033	1.85 (0.62–5.52)	0.2700
Tumor grade Low/1 + Intermediate/2 High/3	1.51 (0.49–4.67)	0.4782	1.19 (0.33–4.38)	0.7886
Tumor diameter ≤2 cm (T1) >2 cm to ≤5 cm (T2)	3.10 (1.15–8.33)	0.0712	3.06 (1.06–8.83)	0.0385
Nodal status No regional lymph nodes metastasis Axillary lymph node metastasis	1.47 (0.51–4.23)	0.4758	1.26 (0.40–4.02)	0.6963
TNM staging IA IIA + IIB	2.42 (0.84–6.96)	0.1018	1.88 (0.63–5.61)	0.2586
Intrinsic subtype Luminal A Other	2.81 (1.02–7.74)	0.0454	2.16 (0.75–6.22)	0.1548
Histological type Invasive ductal carcinoma Invasive lobular carcinoma	0.40 (0.05–3.01)	0.3717	0.47 (0.06–3.56)	0.4611
Ki67 proliferation rate <20% >20%	1.94 (0.73–5.19)	0.1842	1.93 (0.68–5.52)	0.2180
Type of surgery Mastectomy Modified radical mastectomy Breast-conserving surgery	0.59 (0.13–2.68) 1.48 (0.25–8.89	0.1886 0.3456	0.48 (0.10–2.24) 1.47 (0.24–8.80)	0.1044 0.2852
Irradiation + −	2.57 (0.89–7.40)	0.0808	2.19 (0.69–7.01)	0.1846
Brachytherapy + −	3.20 (1.03–9.94)	0.0438	3.87 (1.08–13.9)	0.0377
Chemotherapy + −	1.14 (0.42–3.05)	0.7991	1.17 (0.41–3.37)	0.7702
Endocrine therapy + −	1.77 (0.57–5.51)	0.3206	2.13 (0.67–6.79)	0.2023
HER2+-targeted therapy + −	1.35 (0.72–6.23)	0.2510	3.16 (0.82–8.25)	0.1380

BMI: body mass index; Ki67: proliferation marker; HR: hazard rate ratio; Cl: confidence interval. Other intrinsic subtypes included luminal B, non-luminal HER2+, and triple-negative tumors; underlined *p*-values denote significant differences.

**Table 7:** Multivariate Cox regression analysis assessing the prognostic variables.

Multivariate				
Variables	Disease-Free Survival		Overall Survival	
	Hazard Ratio (HR) (95% CI)	*p*-Value	Hazard Ratio (HR) (95% CI)	*p*-Value
Intrinsic subtype Luminal A Other	3.09 (1.12–8.53)	0.0293	-	-
Brachytherapy + −	3.51 (1.13–10.91)	0.0302	3.68 (1.03–13.23)	0.0457
Tumor diameter ≤2 cm (T1) >2 cm to ≤5 cm (T2)	-	-	2.89 (1.00–8.35)	0.0498

HR: hazard rate ratio; Cl: confidence interval; Other intrinsic subtypes included luminal B, non-luminal HER2+, and triple-negative tumors; underlined *p*-values denote significant differences. The multivariate Cox regression model was adjusted for insignificant features.

## Data Availability

The data presented in this study are available in this article.
